# Structural and functional insights into sorting nexin 5/6 interaction with bacterial effector IncE

**DOI:** 10.1038/sigtrans.2017.30

**Published:** 2017-06-30

**Authors:** Qingxiang Sun, Xin Yong, Xiaodong Sun, Fan Yang, Zhonghua Dai, Yanqiu Gong, Liming Zhou, Xia Zhang, Dawen Niu, Lunzhi Dai, Jia-Jia Liu, Da Jia

**Affiliations:** 1 Department of Pathology, State Key Laboratory of Biotherapy, West China Hospital, Sichuan University and Collaborative Innovation Center of Biotherapy, Chengdu, China; 2 Key Laboratory of Birth Defects and Related Diseases of Women and Children of MOE, Department of Paediatrics, West China Second University Hospital, Sichuan University, Chengdu, China; 3 Department of Pharmacology, West China School of Preclinical and Forensic Medicine, Sichuan University, Chengdu, China; 4 State Key Laboratory of Molecular Developmental Biology, Institute of Genetics and Developmental Biology, Chinese Academy of Sciences, Beijing, China

## Abstract

The endosomal trafficking pathways are essential for many cellular activities. They are also important targets by many intracellular pathogens. Key regulators of the endosomal trafficking include the retromer complex and sorting nexins (SNXs). *Chlamydia trachomatis* effector protein IncE directly targets the retromer components SNX5 and SNX6 and suppresses retromer-mediated transport, but the exact mechanism has remained unclear. We present the crystal structure of the PX domain of SNX5 in complex with IncE, showing that IncE binds to a highly conserved hydrophobic groove of SNX5. The unique helical hairpin of SNX5/6 is essential for binding, explaining the specificity of SNX5/6 for IncE. The SNX5/6–IncE interaction is required for cellular localization of IncE and its inhibitory function. Mechanistically, IncE inhibits the association of CI-MPR cargo with retromer-containing endosomal subdomains. Our study provides new insights into the regulation of retromer-mediated transport and illustrates the intricate competition between host and pathogens in controlling cellular trafficking.

## Introduction

Anterograde and retrograde vesicle trafficking is fundamental for a wide range of cellular processes.^1,2^ In the anterograde trafficking pathway, newly synthesized proteins are folded, post-translationally modified and delivered to either the plasma membrane or to intracellular compartments. Retrograde routes, on the other hand, return membrane-associated components from endosomes to the Golgi apparatus and endoplamic reticulum. Both routes are critical for maintaining organelle identity, lipid homeostasis and many other cellular functions.^3–5^ Deregulation of these processes has been linked with many human diseases including cancer and neurodegeneration.^3–7^

One key component that controls trafficking routes from the endosomal compartment is the retromer complex, which mediates the trafficking from endosomes to the trans-Golgi network (TGN) or the plasma membrane.^3–5^ The core of the retromer complex is an evolutionary conserved trimer consisting of VPS35, VPS26 and VPS29, which recognizes specific endosomal cargo proteins.^8^ Studies in the past have identified many critical partners and regulators of retromer, including TBC1d5,^9,10^ the WASH/actin regulatory complex^11–14^ and dynactin p150^glued^.^15–17^ Each of them plays an essential but distinct role in the formation of retromer-coated structures and retromer-dependent transport. The largest and most well-characterized protein family controlling retromer activity is the sorting nexin (SNX) family, which is characterized by the presence of phox homology (PX) domains.^18,19^ Whereas PX domain is historically regarded as a phosphatidylinositol-3-monophosphate-binding domain, recent studies reveal that PX domains can interact with other lipids and proteins.^18^ So far, three groups of SNX proteins have been shown to function together with retromer, (1) the dimer of SNX1 or SNX2 with SNX5 or SNX6, with all proteins having an additional BAR domain that can sense or induce membrane curvature;^20^ (2) the PX domain-only SNX3;^21,22^ (3) SNX27, a protein harboring discs-large homologous regions and FERM (F for 4.1 protein, E for ezrin, R for radixin and M for moesin) domains in addition to the PX domain.^23,24^ Interestingly, both SNX3 and SNX27 have been shown to directly recognize protein cargo, either on their own (SNX27) or in cooperation with retromer (SNX3).^22,25^ In contrast, it remains to be determined whether SNX1/2/5/6 plays a similar role in retromer-mediated trafficking.

Recent studies have identified the retrograde transport pathway as an important target by pathogenic intracellular bacteria.^26^ Chlamydia, Legionella and many other bacteria subvert this pathway through distinct strategies to promote their intracellular survival and replication. For instance, chlamydiae, a leading cause of human respiratory, genital tract and blinding eye infections, form a unique membrane-bound compartment within host cells, known as inclusion, and replicate within the compartment. *Chlamydia trachomatis* utilize a large number of effector proteins localized on the inclusion membrane to interact with host proteins and to interfere with normal host pathways. One of the effector proteins is IncE, which specifically interacts with SNX5/6 via their PX domain.^27^ SNX5/6 and other retromer components restrict *Chlamydia trachomatis* infection since their depletion leads to enhanced infection through mechanisms that are currently unclear.^27^ Overexpression of IncE in mammalian cells inhibits retromer- and SNX5/6-mediated endosomal trafficking.^27^ Thus, IncE functions to prohibit the inhibition effect exerted by the host retromer components. These studies, however, also raised a few important questions: (1) what is the molecular basis of the interaction between SNX5/6 and IncE? (2) Why does IncE specifically interact with SNX5/6 among many PX domain proteins? (3) How does IncE prohibit SNX5/6- and retromer-mediated transport? Here we have used a combination of structural, biochemical and cellular studies to address these questions.

## Results

### Overall structure of IncE in complex with the PX domain of SNX5 (PX5)

**Figure 1:**
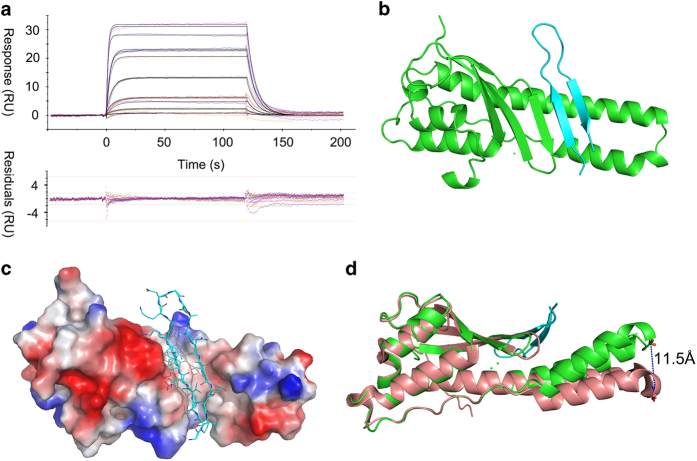
Crystal structure of IncE^109–132^:PX5 complex. (**a**) SPR experiment flowing IncE^109–132^peptide over cell immobilized with PX5 protein. Upper panel shows the reference-subtracted concentration-dependent-binding response and 1:1 fitting model. Bottom panel shows the residual plots for the quality of fitting. (**b**) Crystal structure of PX5 (green) in complex with IncE^109–132^ (cyan). (**c**) IncE (ribbon and line representation) is docked into a hydrophobic groove on PX5 (electrostatic surface representation). The viewing angle in **b** is slightly rotated here to better illustrate the groove. (**d**) Overlay of PX5:IncE^109–132^with structure of apo PX5(PDB:3HPC). The dash line shows the movement of the helical hairpin tip. The view is set by rotating **b** around the horizontal line by 90° clockwise.

Previously, it was reported that the C-terminal 101–132 residues of *Chlamydia trachomatis* IncE interact with SNX5 and SNX6’s PX domain.^27^ We found that a shorter fragment of IncE (109–132) retained the ability to bind to the PX domain of SNX5 (PX5, Supplementary Figure S1A). The dissociation constant (*K*_d_) between IncE^109–132^ and PX5 was measured to be 0.54 μM by surface plasmon resonance (SPR) (Figure 1a). IncE^109–132^ co-elutes with PX5 in a gel filtration column, albeit having very different molecular weights (3 kD vs 20 kD, Supplementary Figure S1B).

To understand this interaction in more detail, we solved the crystal structure of the PX5:IncE^109–132^ complex (Figure 1b). The protein crystallized in *P*1 space group, with two SNX5:IncE dimers per asymmetric unit. The structure was refined to 1.9 Å with a final *R*_free_ of 0.213 (Supplementary Table S1). The resolved model contains residues 28–176 from PX5 (one copy of the PX5 residues 110–120 not modeled due to poor density) and residue 111–131 from IncE. The omit map of IncE peptide shows that the model is well supported by electron density (Supplementary Figure S2). The two non-crystallographic symmetry-related copies are almost identical, with 0.23 Å of root-mean-square deviation (r.m.s.d.) for all atoms aligned. In the complex structure, IncE binds to a hydrophobic groove on PX5 (Figure 1c). IncE forms an anti-parallel β-sheet, which extends the three anti-parallel β-sheets formed by residues 36–62 of PX5. A long protruding double helix in PX5, together with its β strand residues 36–40, forms the hydrophobic groove for IncE binding (Figure 1c). The globular domain of PX5 is similar to the previous published apo structure (0.49 Å r.m.s.d.; Figure 1d).^28^ However, the protruding long double helix is bent toward the IncE-binding site, with the largest movement being 11.5 Å for Cα of Ser115, which is located on the tip of the double helix (Figure 1d).

### Detailed interactions between IncE and PX5

**Figure 2:**
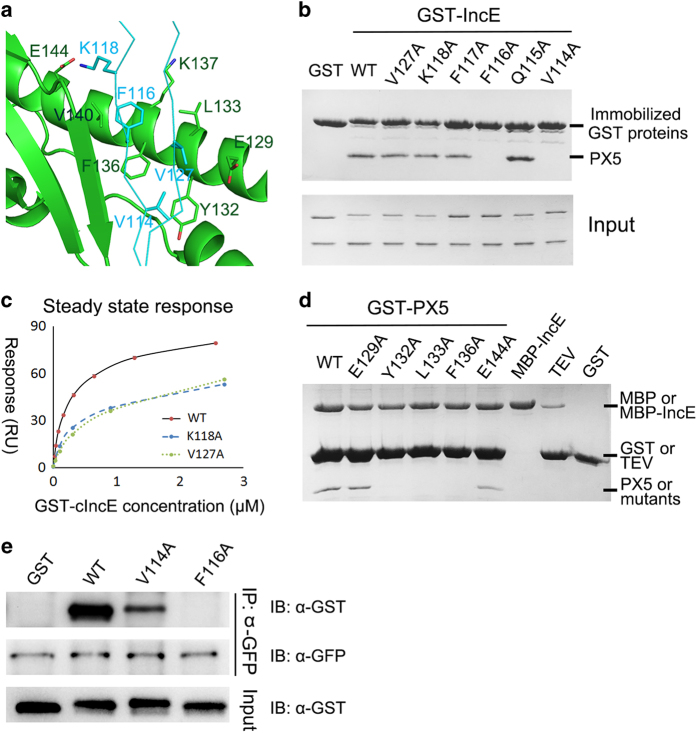
The IncE:PX interface. (**a**) Zoom-in view of interactions between PX5 and IncE from the crystal structure. For clarity, IncE is shown in ribbon representation. Possible interacting (except hydrogen bonds) residues are shown in stick representation. (**b**) Glutathione S-transferase (GST) pull down of GST-IncE^109–132^ WT and mutants with purified PX5 protein. (**c**) SPR steady state analysis of GST-IncE^109–132^ WT and two mutants binding to immobilized PX5. (**d**) MBP pull down of MBP-IncE^109–132^ with GST-PX5 WT and mutants. Before running SDS-PAGE, the samples are digested with excess of tobacco etch virus to allow visualization of bound PX5 protein. (**e**) Immunoprecipitation of green fluorescent protein (GFP)-SNX6 using GFP antibody with purified GST-IncE WT and two mutants.

IncE interacts with PX5 through an extensive network of hydrogen bonds and hydrophobic interactions, resulting in a buried surface area of 646 Å^2^ (Figure 2a and Supplementary Figure S3). All inter-molecular hydrogen bonds are formed between mainchain atoms of the β strands composed by PX5 36–40 and IncE 113–118. E144^PX5^ forms a salt bridge with K118^IncE^. However, the density of Lys118 side chain is rather weak. PX5 presents a hydrophobic floor by the side chains of L133, F136, V140 and the aliphatic side chain portions of K137 and Y132, which forms hydrophobic interaction with IncE residues V114, F116 and V127 (Figure 2a). It is worth mentioning that the distance between V127 and PX5 hydrophobic atoms are generally above 4 Å, which is the upper limit for carbon–carbon hydrophobic interaction.

Next, we individually mutated aforementioned residues of IncE and tested their effect on the interaction with PX5 by pull-down and SPR assays (Figures 2b–d). V114 and F116 of IncE form intimate hydrophobic interactions with PX5, and their alanine substitutions completely abolished binding with PX5. In contrast, mutations to alanine of the two non-interacting solvent exposed residues Q115^IncE^ and F117^IncE^ did not affect binding. K118A^IncE^ and V127A^IncE^ mutations did not impact binding significantly by pull down; however, their affinity toward PX5 was found to be 2–2.5-fold lower than the wild-type protein (Figure 2c). PX5-binding affinity for IncE WT, K118A and V127A was 0.39, 0.79 and 0.98 μM, respectively. In addition, we tested the interaction between full-length SNX6 (immunopurified from cells) and IncE proteins. Whereas SNX6 robustly immune-precipitated wild-type IncE, its interaction with V114A was significantly weaker and its binding to F116A was not detectable (Figure 2e).

Conversely, we also made a series of mutations on PX5 and tested their interactions with IncE. Y132, L133 and F136 form the hydrophobic floor for IncE binding and interact with V114 and F116 of IncE. Mutation of these hydrophobic residues in PX5 (Y132A, L133A or F136A) abolishes its binding with IncE (Figure 2d). E144^PX5^ forms a salt bridge with K118^IncE^, and E144A^PX5^ weakly attenuates the binding. E129^PX5^ forms a side of the groove but is over 4 Å from any residues of IncE; consequently, E129A^PX5^ mutation does not affect binding by pull down. In summary, Y132, L133, F136 of PX5 and V114, F116 of IncE are the most critical residues for maintaining the interaction.

### Specificity of the SNX5/6 interaction with IncE

**Figure 3:**
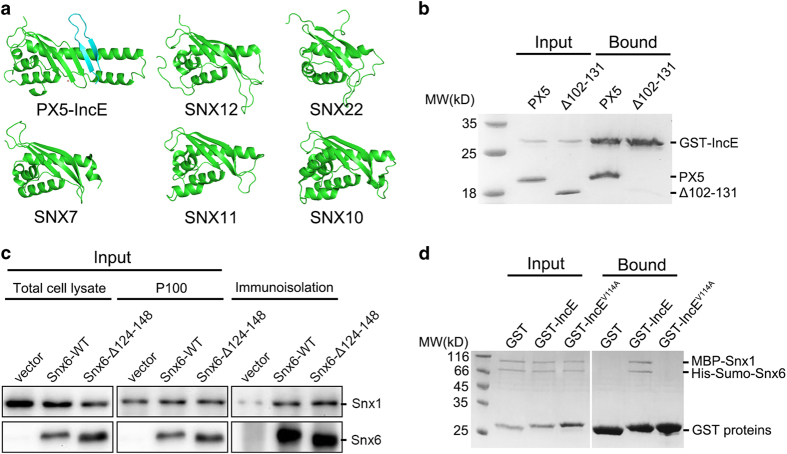
SNX5’s unique protruding helical hairpin is essential for IncE binding. (**a**) Structural comparison of PX5 with PX domains. IncE is colored in cyan. Note that PX5 has a unique long helical hairpin. (**b**) GST pull down of PX5 WT and Δ102–131 mutant with immobilized GST-IncE^109–132^. (**c**) SNX6 WT and Δ124–148 co-immunoprecipitated with SNX1. HEK293T cells were transfected with empty vector, or Flag-YFP-SNX6 constructs, p100 fractions were prepared, and immunopurified with anti-Flag antibody. Lysates were immunoblotted with anti-SNX1 or anti-Flag antibodies. (**d**) GST pull down of purified SNX1/SNX6 protein complex with immobilized GST, GST-IncE^109–132^ WT or V114A.

Although the previous study revealed that IncE interacts with SNX5/6, but not SNX1/2, it remains unclear where the specificity comes from.^27^ The IncE-binding residues are highly conserved among SNX5, SNX6 and closely related SNX32,^19^ including residues 36–40, 132, 133, 136, 140, 144 of PX5, but are varied in other SNX proteins including SNX1, SNX2, SNX3 and SNX27 (Supplementary Figure S4). Since residues 36–40^PX5^ contact IncE only through mainchain atoms and other SNX proteins have a similar β strand in the same region, it is unlikely that these residues provide the specificity for the binding. On the other hand, sequence alignment and structural comparisons show that SNX5/6/32 contain extra residues (D100–V135 in SNX5 and D113–I148 in SNX6) in the PX domain compared with SNX1 and SNX2, which form a unique long helical hairpin (Figure 3a and Supplementary Figure S4).^28^ Many residues in the long hairpin form the base and side of the groove to accommodate IncE strands, in addition to providing key residues to interact with the first strand of IncE. To test the importance of this extended double helix, we deleted part of the double helix containing residues P102–E131. Though the deleted portion does not contain any residues directly contacting IncE, Δ102–131^PX5^ completely abolished the binding between PX5 and IncE (Figure 3b). In contrast, deletion of the similar region in the double helix did not impair the interaction between SNX6 and SNX1 (Figure 3c). In agreement with these data, IncE WT, but not V114 A, retained the ability to interact with the SNX1/SNX6 complex (Figure 3d). Therefore, the unique protruding helical hairpin of SNX5/6 is necessary for IncE binding, but not important for interacting with SNX1.

### Interaction between SNX5/6 and IncE is highly conserved

**Figure 4:**
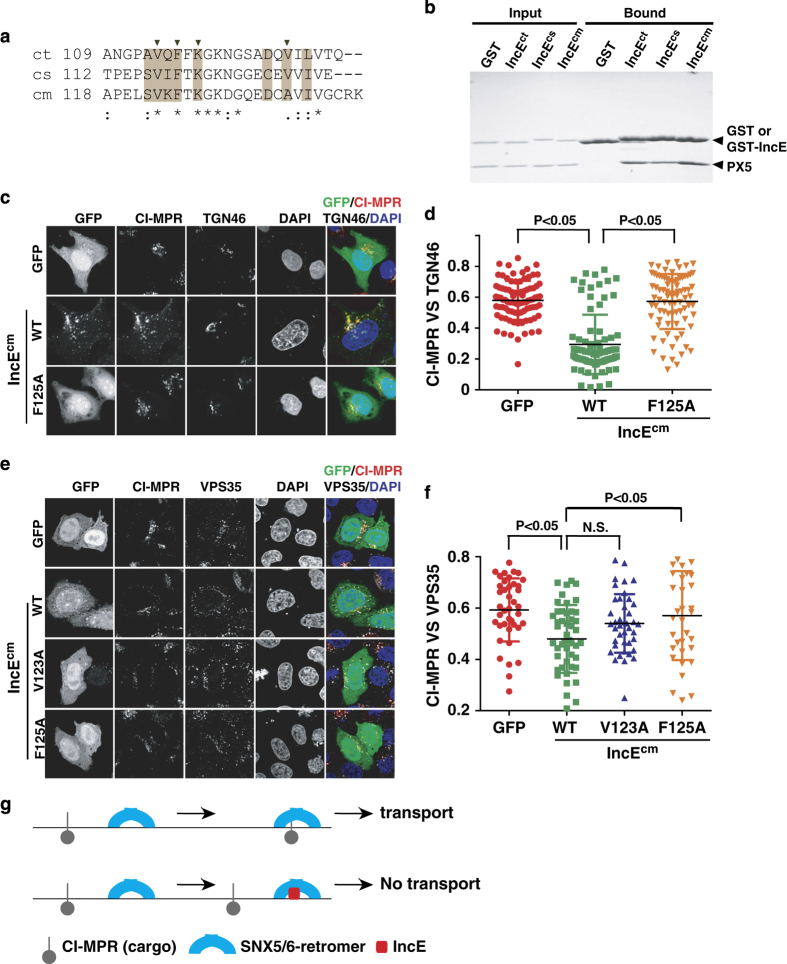
Interaction between IncE and SNX5/6 is conserved, and IncE inhibits CI-MPR loading to retromer vesicles and transport to TGN. (**a**) Sequence alignment of the C terminus of IncE from *Chlamydia* species, *Chlamydia trachomatis* (ct), *Chlamydia suis* (cs) or *Chlamydia muridarum* (cm). Multiple PX sequences are aligned using ClustalW (* for invariant, for conserved, for less conserved changes). Residues at the interface are highlighted in machaccino. Residues whose mutation disrupt or weaken the binding in Figure 2 are labeled with black triangles on top. (**b**) GST pull down with GST or GST-IncE with purified 5PX. (**c**) HeLa cells were transfected with GFP, or GFP-IncE^cm^ WT, and F125A (green), and then fixed and labeled with anti-CI-MPR (red) and TGN46 (white) antibodies. (**d**) Quantitation of CI-MPR co-localization with TGN46 in cells expressing GFP, or various GFP-IncE^cm^. Each dot represents Pearson’s correlation coefficients from one cell. *P*-values shown are the result of one-way ANOVA, *post hoc* Tukey’s test. (**e**) Subcellular localization CI-MPR, VPS35 and IncE^cm^. HeLa cells were transfected with GFP, or various GFP-IncE^cm^ WT, V123A and F125A (green), and then fixed and labeled with anti-CI-MPR (red) and VPS35 (white) antibodies. (**f**) Quantitation of CI-MPR co-localization with VPS35 in cells expressing GFP, or various GFP-IncE^cm^. Each dot represents Pearson’s correlation coefficients from one cell. *P*-values shown are the result of one-way ANOVA, *post hoc* Tukey’s test. (**g**) A model of controlling SNX5/6-mediated transport by effector protein IncE. IncE binds to SNX5/6 and inhibits the loading of CI-MPR to SNX5/6-retromer-coated endosomal structures.

Sequence alignment of IncE from closely related *Chlamydia* species, *Chlamydia trachomatis*, *Chlamydia suis* or *Chlamydia muridarum* shows that the three critical residues, V114, F116 and K118, are strictly conserved, while non-critical ones, Q115 and F117, are varied (Figure 4a). Indeed, we found that the PX domain of SNX5 (PX5) bound to all three IncE isoforms (Figure 4b). Since IncE from *Chlamydia muridarum* displayed stronger co-localization with VPS35 than its paralog from *Chlamydia trachomatis*, we therefore used the former IncE (IncE^cm^) for all cellular studies. IncE^cm^ WT nicely co-localized with VPS35, and its F125A mutant (corresponding to F116A in *Chlamydia trachomatis*, ct) completely lost endosomal localization and became diffusely localized through the cell (Supplementary Figure S5). Consistent with a reduced affinity toward SNX5, the V123A mutant (corresponding to V114A in ct) displayed partial vesicle localization (Supplementary Figure S5). Thus, the SNX5/6:IncE interaction is critical for the subcellular localization of IncE. This interaction could be a general conserved mechanism utilized by multiple *Chlamydia* species during infection.

### Possible mechanism of inhibition of SNX5/6-depedent transport by IncE

To examine the effect of IncE on SNX5/6-mediated cargo transport, we chose to study CI-MPR, a model cargo protein that depends on retromer and SNX5/6 for its transport from endosomes to the TGN (Figures 4c and d).^29,30^ In control cells, CI-MPR displays a compact localization and predominately localizes on the juxtanuclear TGN46-positive compartment, which corresponds to the TGN. Consistent with the published data,^27^ we found that overexpression of IncE^cm^ WT suppressed the transport of CI-MPR and decreased co-localization between CI-M6PR and TGN46. In contrast, the IncE^cm^ F125A mutant, which is defective in its interaction with SNX5, did not interfere with the CI-MPR transport (Figures 4c and d).

Mechanistically, multiple steps are required for the retromer-mediated transport of cargo proteins from endosomes to TGN: (1) recruitment of the retromer complex to cargo-carrying endosomal membrane, (2) cargo enrichment to retromer-containing endosomal subdomains, (3) membrane tubulation and scission, (4) transport of newly formed vesicles and (5) tethering to the TGN.^4,31,32^ Since IncE does not affect the first step of the process, endosomal recruitment of retromer and SNX5/6, to address the mechanism of IncE, we began to examine the second step by determining co-localization of CI-MPR and VPS35. Overexpression of IncE^cm^ caused a decrease in CI-MPR co-localization with VPS35 (Figures 4e and f). This ability of IncE to inhibit the co-localization between CI-MPR and VPS35 seems to correlate with the affinity between SNX5/6 and IncE as IncE^cm^ V123A, which still retains some affinity toward SNX6, only partially reduced the co-localization. Thus, IncE may inhibit retromer-mediated transport by decreasing the association of cargo with retromer.

## Discussion

During infection, *Chlamydia* secretes a large number of effector proteins, which in turn interact with host proteins to facilitate bacterial survival and replication.^27^ IncE directly interacts with SNX5/6 and inhibits SNX5/6 and retromer-dependent transport. Depletion of SNX5/6 facilitates *Chlamydia* infection, thus SNX5/6 function as a restriction factor and IncE functions to suppress this inhibition. To understand the interaction between IncE and SNX5/6, we performed biochemical, structural and cellular studies. In the process, we were able to show that IncE binds to a highly conserved hydrophobic groove of SNX5/6. The interaction between SNX5/6 and IncE is conserved among several *Chlamydia* species. Several key residues for binding to SNX5/6 identified by our crystal structure and biochemical assays are strictly conserved, suggesting that the SNX5/6–IncE contact is likely a conserved feature of Chlamydia–host interaction and may play an important function during Chlamydia infection.

The beauty of studying host–pathogen interaction is that it can not only provide us new knowledge on pathogen survival and infection, but uncover novel information on host biology. We showed that IncE inhibits retromer transport through its direct interaction with SNX5/6, and its inhibitory effect correlates with its ability to reduce the co-localization between cargo and retromer. During the final stages that our manuscript is under review, two papers reporting the interaction between SNX5/6–IncE were published, consistent with our findings in general.^33,34^ Elwell *et al.* found that SNX5 associates with CI-MPR biochemically, and IncE peptide inhibits the interaction. The biochemical studies, together with our cellular observations, suggest a simple model: SNX5/6 may contribute to recognition of the CI-MPR cargo (and other cargo proteins) by the retromer. The addition of IncE prevents the association between SNX5/6 and CI-MPR, and the enrichment of CI-MPR to retromer-containing endosomal domains (Figure 4g). Recently, both SNX3 and SNX27 have been found to bind to retromer cargo directly,^22,25^ and future studies will be necessary to test whether SNX5/6 directly interacts with CI-MPR. In conclusion, our study elucidates the molecular basis for the SNX5/6 interaction with the effector protein IncE, and suggests that IncE may function to disrupt the association between protein cargo and retromer as a mechanism to control retromer transport.

## Materials and methods

### Antibodies and plasmids

Antibodies used in this study are listed in Supplementary Table S2. All constructs were generated by standard PCR and confirmed by DNA sequencing.

### Cell culture, immunofluorescent staining and confocal microscopy

HEK293T and HeLa cells were maintained in Dulbecco’s modified Eagles medium (Hyclone, GE Helthcare, Little Chalfont, UK) supplemented with 10% (vol/vol) fetal bovine serum (Sangon Biotech, Shanghai, China) at 37 °C incubator supplied with 5% CO_2_. HEK293T cells were transfected with the plasmid DNA using the standard calcium phosphate transfection protocol, and HeLa cells were transfected with TurboFect transfection reagent (Thermo Scientific, Waltham, MA, USA). Confocal images were acquired by the Olympus FV-1000 confocal microscope (Shinjuku, Tokyo, Japan) and the Zeiss LSM 780 confocal microscope (Oberkochen, Germany) and were analyzed using NIH ImageJ software.^35^ All cellular experiments were duplicated at least once.

### Immunoprecipitation

Immunoprecipitations were prepared and analyzed as described.^9,16,36,37^ Briefly, transiently transfected HEK293T cell lysates were prepared in lysis buffer (50 mM Tris-HCl, 150 mM NaCl, 1 mM EDTA and 0.5% (vol/vol) NP-40) supplemented with cocktail protease inhibitors (biotool, Jupiter, FL, USA) on ice for 30 min and centrifuged at 16 000 *g* for 15 min at 4 °C. The supernatant was incubated with anti-GFP antibodies at 4 °C for 30 min. Protein A/G PLUS-Agarose beads (Santa Cruz, Dallas, TX, USA, sc-2003) were washed twice with lysis buffer and were added in to the supernatant with 50 mg GST-IncE proteins. After overnight incubation, the precipitates were washed five times in lysis buffer. The 2× SDS-PAGE loading buffer was added into the washed precipitates to elute the samples. The elution was subjected to SDS-PAGE and immunoblotting after boiled at 98 °C for 10 min. Immunoblots were imaged with the ChemiDoc Touch Imaging System (Bio-Rad, Hercules, CA, USA).

### Protein expression and purification

The mouse SNX5 PX domain (residues 20–180, PX5) or C terminus residues 109–132 of IncE was cloned into a pGEX-4T1-based expression vector incorporating a tobacco etch virus-cleavable N-terminal His-tag fusion. The plasmid was transformed into *Escherichia coli* BL-21 (DE3) and grown in LB broth medium. Expression of protein was induced by the addition of 0.5 mM isopropyl β-D-1-thiogalactopyranoside and the culture was grown overnight at 20 °C. Cells were collected and sonicated in lysis buffer (20 mM Tris pH 8.0, 200 mM NaCl and 1 mM PMSF). PX5 was purified on a GST column and eluted after tobacco etch virus cleavage in buffer containing: 20 mM Tris pH 8.0, 200 mM NaCl, 1 mM EDTA and 2 mM BME, followed by a Superdex 200 increase gel filtration column on the Äkta Pure (GE Healthcare, Little Chalfont, UK) using the gel filtration buffer (20 mM Tris pH 8.0, 200 mM NaCl, 2 mM BME). Eluted PX5 was concentrated to 8 mg ml^−1^ and frozed at −80 °C. GST-IncE^109–132^ was eluted with 20 mM Tris pH 8.0, 200 mM NaCl, 1 mM EDTA, 2 mM BME and 10 mM reduced glutathione. MBP-IncE^109–132^ was cloned into a modified pMal-5x vector containing an additional His6 tag and tobacco etch virus-cleavable site. The protein was expressed similarly and purified by nickel beads, eluted with 20 mM Tris pH 8.0, 200 mM NaCl, 2 mM BME and 300 mM imidazole.

### Crystallization and data collection

IncE^109–132^:PX5 complex is formed by mixing threefold molar excess of IncE peptide with purified PX5 protein and followed by crystal screening in 96-well plate using Gryphon robots (Art Robbins Instruments, LLC, Sunnyvale, CA, USA). Initial crystallization conditions were identified from the Crystal Screen I (Hampton Research, Aliso Viejo, CA, USA) using hanging-drop vapor-diffusion methods at 18 °C by mixing equal amounts of protein solution (5 mg ml^−1^) and reservoir solution containing 0.2 M ammonium acetate, 0.1 M sodium acetate pH 4.6, 30% w/v polyethylene glycol 4000. The tiny crystal was further optimized in 0.1 M ammonium acetate, 0.1 M sodium acetate pH 4.6, 32% w/v polyethylene glycol 3350. Rod-shaped crystal with diameter of about 20 nm was obtained after about 3 days. An amount of 15% (v/v) glycerol was supplemented with crystallization condition as the cryo-protectant. X-ray diffraction data were collected at Shanghai Synchrotron Radiation facility beamline BL17U1.^38^ The data collection statistics are given in Supplementary Table S1.

### Structure solution and refinement

The structure of PX5:IncE^109–132^ complex was solved using the molecular replacement method with the program MolRep^39^ with the coordinates of *Rattus norvegicus* SNX5 PX domain (pdb code: 3HPC) as the search model. In each asymmetric unit, two PX5 molecules were identified. After several refines, the resulting electron density was examined in the program COOT^40^ and allowed modeling of the two IncE peptides containing amino-acid Gly111 to Thr131. Several cycles of map fitting and refinement using the program Refmac5^(ref. 41)^ led to convergence. Translation/Libration/Screw refinement and non-crystallographic symmetry restraints were used in the refinement process.^42^ Data in the interval 50.00–1.90 Å resolution was used and at the end of the refinement the *R*-value was 0.167 (*R*_free_=0.213) for all reflections.

### SPR

SPR experiments were performed with Biacore T100 (Biacore AB, Uppsala, Sweden). PX5 protein (50 ng μl^−1^, 110 μl, in 5 mM sodium citrate pH 5.5) was immobilized onto a CM 5 Chip as per the manufacturer’s recommendations. The inactivated PX domain (by 1 min flow of 10 mM H_2_SO_4_) was used in reference cell. The running buffer consisted of 20 mM Na HEPES pH 7.5, 200 mM NaCl, 0.005% Triton-X100. Experiments were performed at 25 °C. Different concentrations of peptides (33 nM–2.7 μM in running buffer) were applied to the chip surface at a flow rate of 10 μl min^−1^. All sensorgrams were processed using automatic correction for non-specific bulk refractive index effects. The equilibrium constant (KD) was determined by the 1:1 Langmuir-binding model or 1:1 steady state analysis provided by the Biacore evaluation software (GE Helthcare, Little Chalfont, UK).

### Pull down

To assess PX5 interaction with IncE C-terminal residues, either PX5 or IncE or their mutants were immobilized on beads with the help of a fusion GST/MBP tag. Excess of soluble binding partner proteins are incubated with the immobilized protein in a total volume of 1 ml for 1 h at 4 °C. After extensive washing with buffer containing 20 mM Tris, pH 8.0, 200 mM NaCl, 0.005% Triton-X100 and 2 mM DTT, bound proteins were separated by SDS/PAGE and visualized by Coomassie staining. Each experiment was repeated at least once and checked for consistency.

### Protein data bank code

Coordinates were deposited with accession codes 5WY2.

## Figures and Tables

**Table 1:** Crystallographic data collection and refinement statistics, related to Figure 1

	*PX5:IncE*
Cell axial lengths (Å)	*a*=37.44, *b*=43.71, *c*=60.07 *α*=94.79, *β*=99.81, *γ*=100.97
Space group	P1
	
*Data collection*	
Resolution range (Å)	50.00–1.90 (1.95–1.90)
Number of observed reflections	55382 (27655)
Number of unique reflections	27655 (1378)
Completeness (%)	96.6 (95.8)
Redundancy	2.0 (2.0)
Highest shell CC*	0.732
Mean I/I_sigma_	12.1 (1.2)
Solvent content (%)	43.5
	
*Refinement*	
Resolution range (Å)	50.00–1.90 (1.95–1.90)
Number of working reflections	27027 (1917)
Number of test reflections	1458 (91)
*R*_work_^a^ (no. of reflections)	0.167 (0.264)
*R*_free_^b^ (no. of reflections)	0.213 (0.286)
r.m.s.d. bond lengths (Å)	0.008
r.m.s.d. bond angles (°)	1.263
	
*Average B-factors (Å2) (# of atoms)*	
Protein atoms	37.1 (2350)
Inhibitor atoms	51.8 (306)
Waters atoms	42.3 (263)
	
*Ramachandran plot*	
Most favored regions (%)	95.1
Allowed regions (%)	3.5
General allowed regions (%)	0.3
Disallowed regions (%)	1.0

*R*_work_^b^=Σ|Fo−Fc|/|Fo|, where Fc and Fo are the calculated and observed structure factor amplitudes, respectively.

*R*_free_^c^ calculated as for *R*_work_ but for 5.0% of the total reflections chosen at random and omitted from refinement for all data sets.

## References

[bib1] Anitei M, Hoflack B. Bridging membrane and cytoskeleton dynamics in the secretory and endocytic pathways. Nat Cell Biol 2012; 14: 11–19. 10.1038/ncb2409 22193159

[bib2] Guo Y, Sirkis DW, Schekman R. Protein sorting at the trans-Golgi network. Annu Rev Cell Dev Biol 2014; 30: 169–206. 25150009 10.1146/annurev-cellbio-100913-013012

[bib3] Bonifacino JS, Hurley JH. Retromer. Curr Opin Cell Biol 2008; 20: 427–436. 18472259 10.1016/j.ceb.2008.03.009 PMC2833274

[bib4] Burd C, Cullen PJ. Retromer: a master conductor of endosome sorting. Cold Spring Harb Perspect Biol 2014; 6: a016774. 24492709 10.1101/cshperspect.a016774 PMC3941235

[bib5] Seaman MN. The retromer complex—endosomal protein recycling and beyond. J Cell Sci 2012; 125: 4693–4702. 23148298 10.1242/jcs.103440 PMC3517092

[bib6] Small SA, Petsko GA. Retromer in Alzheimer disease, Parkinson disease and other neurological disorders. Nat Rev Neurosci 2015; 16: 126–132. 25669742 10.1038/nrn3896

[bib7] Sun Q, Chen X, Zhou Q, Burstein E, Yang S, Jia D. Inhibiting cancer cell hallmark features through nuclear export inhibition. Signal Transduct Target Ther 2016; 1: 1–10. 10.1038/sigtrans.2016.10 PMC5661660 29263896

[bib8] Seaman MN, McCaffery JM, Emr SD. A membrane coat complex essential for endosome-to-Golgi retrograde transport in yeast. J Cell Biol 1998; 142: 665–681. 9700157 10.1083/jcb.142.3.665 PMC2148169

[bib9] Jia D, Zhang JS, Li F, Wang J, Deng Z, White MA et al. Structural and mechanistic insights into regulation of the retromer coat by TBC1d5. Nat Commun 2016; 7: 13305. 27827364 10.1038/ncomms13305 PMC5105194

[bib10] Seaman MN, Harbour ME, Tattersall D, Read E, Bright N. Membrane recruitment of the cargo-selective retromer subcomplex is catalysed by the small GTPase Rab7 and inhibited by the Rab-GAP TBC1D5. J Cell Sci 2009; 122: 2371–2382. 19531583 10.1242/jcs.048686 PMC2704877

[bib11] Derivery E, Sousa C, Gautier JJ, Lombard B, Loew D, Gautreau A. The Arp2/3 activator WASH controls the fission of endosomes through a large multiprotein complex. Dev Cell 2009; 17: 712–723. 19922875 10.1016/j.devcel.2009.09.010

[bib12] Gomez TS, Billadeau DD. A FAM21-containing WASH complex regulates retromer-dependent sorting. Dev Cell 2009; 17: 699–711. 19922874 10.1016/j.devcel.2009.09.009 PMC2803077

[bib13] Harbour ME, Breusegem SY, Antrobus R, Freeman C, Reid E, Seaman MN. The cargo-selective retromer complex is a recruiting hub for protein complexes that regulate endosomal tubule dynamics. J Cell Sci 2010; 123: 3703–3717. 20923837 10.1242/jcs.071472 PMC2964111

[bib14] Jia D, Gomez TS, Metlagel Z, Umetani J, Otwinowski Z, Rosen MK et al. WASH and WAVE actin regulators of the Wiskott-Aldrich syndrome protein (WASP) family are controlled by analogous structurally related complexes. Proc Natl Acad Sci USA 2010; 107: 10442–10447. 20498093 10.1073/pnas.0913293107 PMC2890800

[bib15] Hong Z, Yang Y, Zhang C, Niu Y, Li K, Zhao X et al. The retromer component SNX6 interacts with dynactin p150(Glued) and mediates endosome-to-TGN transport. Cell Res 2009; 19: 1334–1349. 19935774 10.1038/cr.2009.130

[bib16] Niu Y, Zhang C, Sun Z, Hong Z, Li K, Sun D et al. PtdIns(4)P regulates retromer-motor interaction to facilitate dynein-cargo dissociation at the trans-Golgi network. Nat Cell Biol 2013; 15: 417–429. 23524952 10.1038/ncb2710

[bib17] Wassmer T, Attar N, Harterink M, van Weering JR, Traer CJ, Oakley J et al. The retromer coat complex coordinates endosomal sorting and dynein-mediated transport, with carrier recognition by the trans-Golgi network. Dev Cell 2009; 17: 110–122. 19619496 10.1016/j.devcel.2009.04.016 PMC2714578

[bib18] Teasdale Rohan D, Collins Brett M. Insights into the PX (phox-homology) domain and SNX (sorting nexin) protein families: structures, functions and roles in disease. Biochem J 2012; 441: 39–59. 22168438 10.1042/BJ20111226

[bib19] van Weering JR, Cullen PJ. Membrane-associated cargo recycling by tubule-based endosomal sorting. Semin Cell Dev Biol 2014; 31: 40–47. 24641888 10.1016/j.semcdb.2014.03.015

[bib20] Rojas R, Kametaka S, Haft CR, Bonifacino JS. Interchangeable but essential functions of SNX1 and SNX2 in the association of retromer with endosomes and the trafficking of mannose 6-phosphate receptors. Mol Cell Biol 2007; 27: 1112–1124. 17101778 10.1128/MCB.00156-06 PMC1800681

[bib21] Harrison MS, Hung CS, Liu TT, Christiano R, Walther TC, Burd CG. A mechanism for retromer endosomal coat complex assembly with cargo. Proc Natl Acad Sci USA 2014; 111: 267–272. 24344282 10.1073/pnas.1316482111 PMC3890810

[bib22] Lucas M, Gershlick DC, Vidaurrazaga A, Rojas AL, Bonifacino JS, Hierro A. Structural mechanism for cargo recognition by the retromer complex. Cell 2016; 167: 1623–1635.e14. 27889239 10.1016/j.cell.2016.10.056 PMC5147500

[bib23] Steinberg F, Gallon M, Winfield M, Thomas EC, Bell AJ, Heesom KJ et al. A global analysis of SNX27-retromer assembly and cargo specificity reveals a function in glucose and metal ion transport. Nat Cell Biol 2013; 15: 461–471. 23563491 10.1038/ncb2721 PMC4052425

[bib24] Temkin P, Lauffer B, Jager S, Cimermancic P, Krogan NJ, von Zastrow M. SNX27 mediates retromer tubule entry and endosome-to-plasma membrane trafficking of signalling receptors. Nat Cell Biol 2011; 13: 715–721. 21602791 10.1038/ncb2252 PMC3113693

[bib25] Gallon M, Clairfeuille T, Steinberg F, Mas C, Ghai R, Sessions RB et al. A unique PDZ domain and arrestin-like fold interaction reveals mechanistic details of endocytic recycling by SNX27-retromer. Proc Natl Acad Sci USA 2014; 111: E3604–E3613. 25136126 10.1073/pnas.1410552111 PMC4156734

[bib26] Personnic N, Bärlocher K, Finsel I, Hilbi H. Subversion of retrograde trafficking by translocated pathogen effectors. Trends Microbiol 24: 450–462. 26924068 10.1016/j.tim.2016.02.003

[bib27] Mirrashidi KM, Elwell CA, Verschueren E, Johnson JR, Frando A, Von Dollen J et al. Global mapping of the Inc-human interactome reveals that retromer restricts chlamydia infection. Cell Host Microbe 2015; 18: 109–121. 26118995 10.1016/j.chom.2015.06.004 PMC4540348

[bib28] Koharudin LM, Furey W, Liu H, Liu YJ, Gronenborn AM. The phox domain of sorting nexin 5 lacks phosphatidylinositol 3-phosphate (PtdIns(3)P) specificity and preferentially binds to phosphatidylinositol 4,5-bisphosphate (PtdIns(4,5)P2). J Biol Chem 2009; 284: 23697–23707. 19553671 10.1074/jbc.M109.008995 PMC2749144

[bib29] Arighi CN, Hartnell LM, Aguilar RC, Haft CR, Bonifacino JS. Role of the mammalian retromer in sorting of the cation-independent mannose 6-phosphate receptor. J Cell Biol 2004; 165: 123–133. 15078903 10.1083/jcb.200312055 PMC2172094

[bib30] Wassmer T, Attar N, Bujny MV, Oakley J, Traer CJ, Cullen PJ. A loss-of-function screen reveals SNX5 and SNX6 as potential components of the mammalian retromer. J Cell Sci 2007; 120: 45–54. 17148574 10.1242/jcs.03302

[bib31] Cullen PJ, Korswagen HC. Sorting nexins provide diversity for retromer-dependent trafficking events. Nat Cell Biol 2011; 14: 29–37. 22193161 10.1038/ncb2374 PMC3613977

[bib32] Rojas R, van Vlijmen T, Mardones GA, Prabhu Y, Rojas AL, Mohammed S et al. Regulation of retromer recruitment to endosomes by sequential action of Rab5 and Rab7. J Cell Biol 2008; 183: 513–526. 18981234 10.1083/jcb.200804048 PMC2575791

[bib33] Elwell CA, Czudnochowski N, von Dollen J, Johnson JR, Nakagawa R, Mirrashidi K et al. Chlamydia interfere with an interaction between the mannose-6-phosphate receptor and sorting nexins to counteract host restriction. Elife 2017; 6: e22709. 28252385 10.7554/eLife.22709 PMC5364026

[bib34] Paul B, Kim HS, Kerr MC, Huston WM, Teasdale RD, Collins BM. Structural basis for the hijacking of endosomal sorting nexin proteins by Chlamydia trachomatis. Elife 2017; 6: e22311. 28226239 10.7554/eLife.22311 PMC5348129

[bib35] Abramoff MD, Magalhaes PJ, Ram SJ. Image Processing with ImageJ. Biophotonics International 2004; 11: 36–42.

[bib36] Jia D, Gomez TS, Billadeau DD, Rosen MK. Multiple repeat elements within the FAM21 tail link the WASH actin regulatory complex to the retromer. Mol Biol Cell 2012; 23: 2352–2361. 22513087 10.1091/mbc.E11-12-1059 PMC3374753

[bib37] Niu Y, Dai Z, Liu W, Zhang C, Yang Y, Guo Z et al. Ablation of SNX6 leads to defects in synaptic function of CA1 pyramidal neurons and spatial memory. Elife 2017; 6: e20991. 28134614 10.7554/eLife.20991 PMC5323044

[bib38] Wang Z, Pan Q, Yang L, Zhou H, Xu C, Yu F et al. Automatic crystal centring procedure at the SSRF macromolecular crystallography beamline. J Synchrotron Radiat 2016; 23: 1323–1332. 27787238 10.1107/S160057751601451X

[bib39] Vagin A, Teplyakov A. MOLREP:an automated program for molecular replacement. J Appl Cryst 1997; 30: 1022–1025.

[bib40] Emsley P, Cowtan K. Coot: model-building tools for molecular graphics. Acta Crystallogr D Biol Crystallogr 2004; 60: 2126–2132. 15572765 10.1107/S0907444904019158

[bib41] Murshudov GN, Vagin AA, Dodson EJ. Refinement of macromolecular structures by the maximum-likelihood method. Acta Crystallogr D Biol Crystallogr 1997; 53: 240–255. 15299926 10.1107/S0907444996012255

[bib42] Painter J, Merritt EA. TLSMD web server fpr the generation of multi-group TLS models. J Appl Cryst 2006; 39: 109–111.

