# Supplementary material for: Structural and functional insights into sorting nexin 5/6 interaction with bacterial effector IncE
Source: Signal Transduct Target Ther. 2017 Jun 30;2:17030–. doi: 10.1038/sigtrans.2017.30 (PMC5661634; doi:10.1038/sigtrans.2017.30)
Supplement: Supplementary Information [file sigtrans201730-s1.docx]

**Inventory of the Supplementary Information**

- Figure S1. IncE^109-132^ binds to PX5 in vitro. Related to Figure 1.
- Figure S2. Stereo view of 2Fo-Fc omit map. Related to Figure 1.
- Figure S3. All hydrogen bonds (blue dash line) and salt bridge formed between IncE^109-132^ and PX5. Related to Figure 2.
- Figure S4. Sequence alignment of PX domains from representative organisms and SNX proteins. Related to Figure 3.
- Figure S5. Subcellular localization of IncE from Chlamydia muridarum (IncE^cm^). Related to Figure 4.
- Table S1. Crystallography Data Collection and Refinement Statistics. Related to Figure 1.
- Table S2. Summary of Antibodies Used in this Study. Related to Figure 4.

Fig. S1. IncE^109-132^ binds to PX5 in vitro, related to Figure 1. A) Pull down of PX5 with immobilized GST-IncE fragments. IncE^109-132^ and IncE^101-132^ bind to PX5 at similar levels. B) Left panel: size exclusion chromatography of IncE^109-132^ with PX5. Right panel: SDS-PAGE analysis of pure IncE^109-132^, pure PX5 or the size exclusion fractions (8, 10) from the left panel.

Fig. S2. Stereo view of 2Fo-Fc omit map generated by CCP4, contoured at 1.0 sigma level. Shown is the IncE and PX5 interacting region, with important residues labeled. Related to Figure 1.

Fig. S3. All hydrogen bonds (blue dash line) and salt bridge formed between IncE^109-132^ and PX5, related to Figure 2. The hydrogen bonds are formed between two inter-molecular beta strands. There is one salt bridge formed between K118 and E144.

Fig. S4. Sequence alignment of PX domains from representative organisms and SNX proteins, related to Figure 3. Multiple PX sequences are aligned using ClustalW (* for invariant, : for conserved, . for less conserved changes). Residues at the interface are highlighted in machaccino. Residues whose mutation disrupt or weaken the binding in Figure 2 are labeled with black triangles on top, and residues deleted in Figure 3 are marked with blue.

Figure S5. Subcellular localization of IncE from Chlamydia muridarum (IncE^cm^), related to Figure 4. A) Subcellular localization of IncE^cm^ wild-type (WT), V123A, and F125A. Hela cells were transfected with GFP, or various GFP-IncE^cm^ (green), and then fixed and labeled with anti-VPS35 antibody (red). B) Quantitation of VPS35 colocalization with IncEcm in cells expressing GFP, or various GFP-IncE^cm^. Each dot represents Pearson’s correlation coefficients from one cell.Subcellular localization CI-MPR, TGN46, and IncE^cm^. P values shown are the result of one-way ANOVA, post hoc Tukey's test.

| Table S1. Crystallographic data collection and refinement Statistics, related to Figure 1. | | |
| --- | --- | --- |
|  |  |  |
|  | ***PX5:IncE*** |  |
|  |  |  |
| Cell axial lengths (Å) | *a*=37.44, *b*=43.71, *c*=60.07  α=94.79, β=99.81, γ=100.97 |  |
| Spacegroup | P1 |  |
|  |  |  |
| **Data collection** |  |  |
| Resolution range (Å) | 50.00-1.90 (1.95-1.90) |  |
| Number of observed reflections | 55382 (27655) |  |
| Number of unique reflections | 27655 (1378) |  |
| Completeness (%) | 96.6 (95.8) |  |
| Redundancy | 2.0 (2.0) |  |
| Highest shell CC* | 0.732 |  |
| Mean I/I_sigma_ | 12.1 (1.2) |  |
| Solvent content (%) | 43.5 |  |
|  |  |  |
| **Refinement** |  |  |
| Resolution range (Å) | 50.00-1.90 (1.95-1.90) |  |
| Number of working reflections | 27027 (1917) |  |
| Number of test reflections | 1458 (91) |  |
| R_work_^a^ (no. of reflections) | 0.167 (0.264) |  |
| R_free_^b^ (no. of reflections) | 0.213 (0.286) |  |
| R.m.s. deviation bond lengths (Å) | 0.008 |  |
| R.m.s. deviation bond angles (°) | 1.263 |  |
|  |  |  |
| **Average B-factors (Å2) (# of atoms)** |  |  |
| Protein atoms | 37.1 (2350) |  |
| Inhibitor atoms | 51.8 (306) |  |
| Waters atoms | 42.3 (263) |  |
|  |  |  |
| **Ramachandran plot** |  |  |
| Most favored regions (%) | 95.1 |  |
| Allowed regions (%) | 3.5 |  |
| General allowed regions (%) | 0.3 |  |
| Disallowed regions (%) | 1.0 |  |

R_work_^b^ = Σ|Fo – Fc|/|Fo|, where Fc and Fo are the calculated and observed structure factor amplitudes, respectively
R_free_^c^ calculated as for R_work_ but for 5.0% of the total reflections chosen at random and omitted from refinement for all data sets

Table S2. Summary of Antibodies Used in this Study, related to Figure 4.

| Antibody | Company | Catalog | Concentration used or dilution fold |
| --- | --- | --- | --- |
| VPS35 | Gift from Dr. Dan Billadeau |  | 1:2000 |
| TGN46 | abcam | ab16059 | 1:1000 |
| CI-MPR | Bio-Rad | MCA2048APC | 1:1000 |
| GFP | Santa Cruz | sc-9996 | 1:500 |
| GST | Santa Cruz | sc-138 | 1:500 |
| SNX1 | Abcam | ab134126 | 1:1000 |
| Flag | sigma-aldrich | F7425 | 1:1000 |
| goat anti-mouse IgG-HRP | Santa Cruz | sc-2005 | 1:2000 |
| TRITC affinipure goat anti-mouse IgG | Jackson ImmunoResearch | 115-025-003 | 0.5 μg/ml |
| TRITC affinipure goat anti-rabbit IgG | Jackson ImmunoResearch | 111-025-003 | 0.5 μg/ml |
| Alexa Fluor 647 affinipure goat anti-mouse IgG | Jackson ImmunoResearch | 115-605-003 | 0.5 μg/ml |
| Alexa Fluor 647 affinipure goat anti-rabbit IgG | Jackson ImmunoResearch | 111-605-003 | 0.5 μg/ml |
