# Supplementary figures and images for: Structural and functional insights into sorting nexin 5/6 interaction with bacterial effector IncE
Source: Signal Transduct Target Ther. 2017 Jun 30;2:17030–. doi: 10.1038/sigtrans.2017.30 (PMC5661634; doi:10.1038/sigtrans.2017.30)

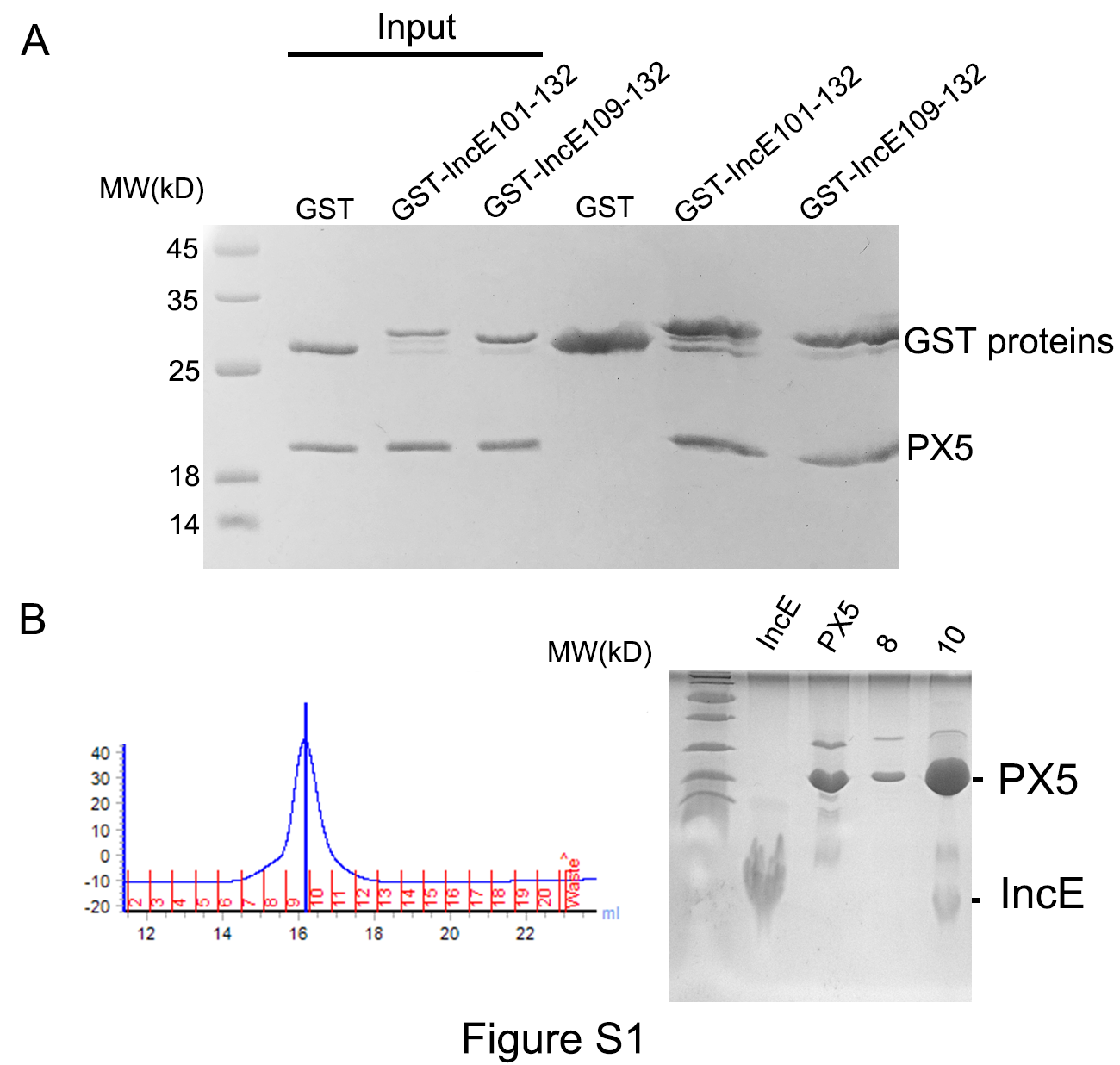

Supplement: Supplementary Figure S1 [file sigtrans201730-s2.tiff]

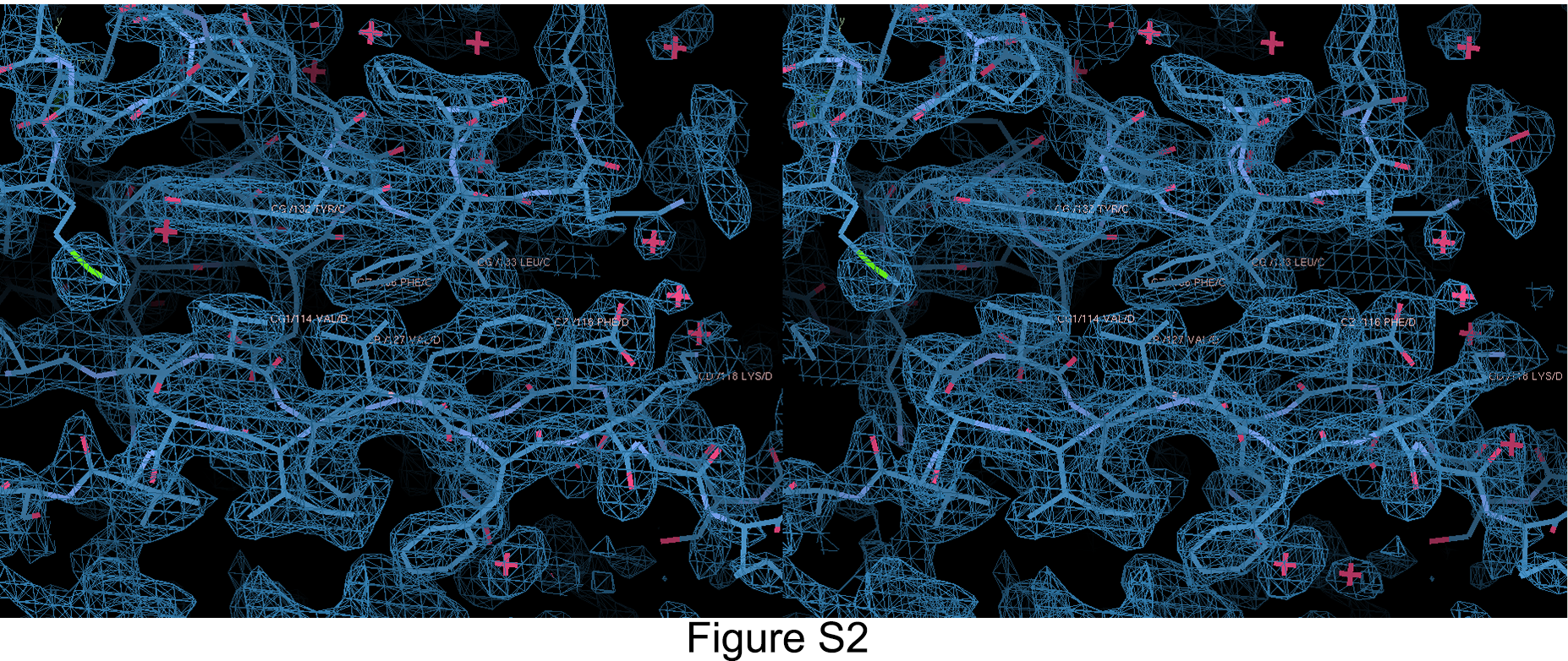

Supplement: Supplementary Figure S2 [file sigtrans201730-s3.tiff]

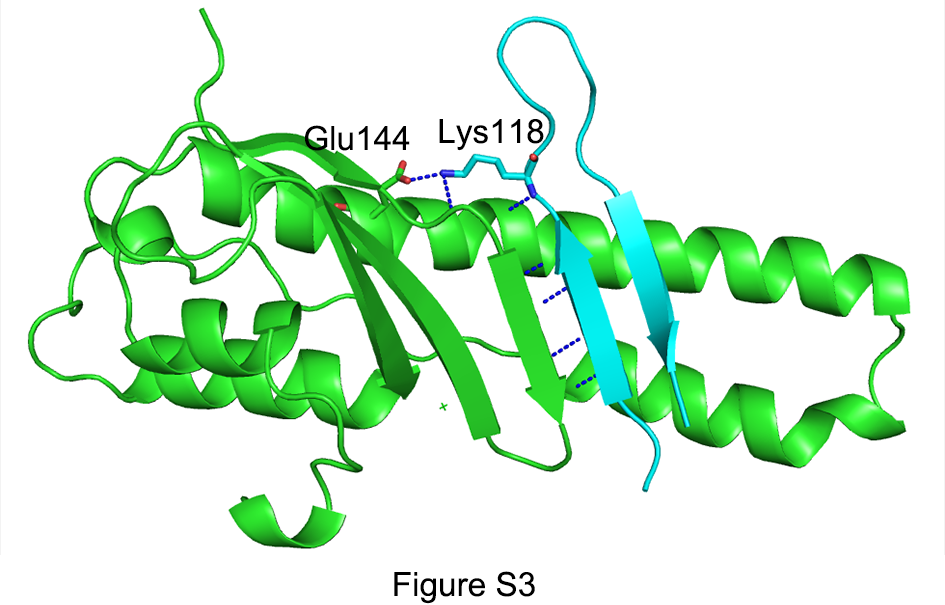

Supplement: Supplementary Figure S3 [file sigtrans201730-s4.tiff]

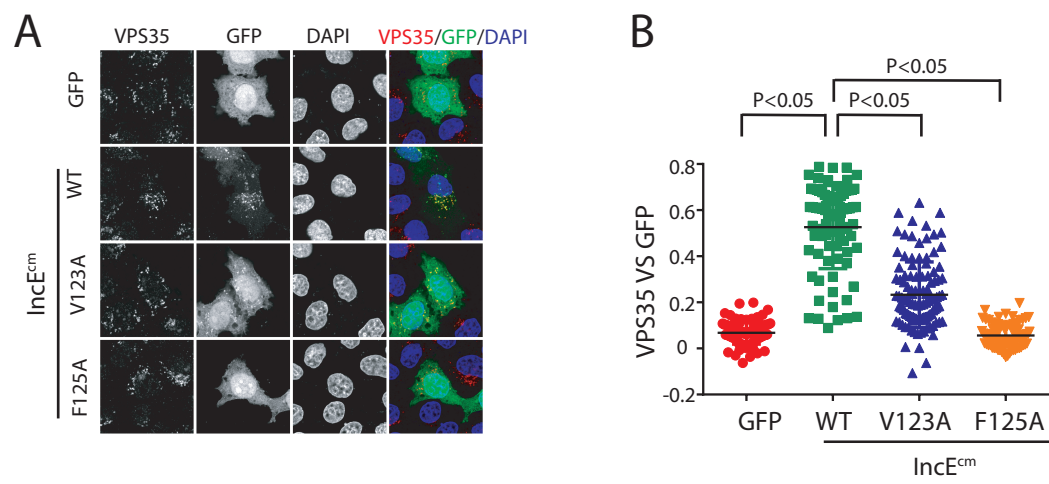

Figure S5

Supplement: Supplementary Figure S5 [file sigtrans201730-s6.pdf]
