# Supplementary material for: Structural and functional insights into sorting nexin 5/6 interaction with bacterial effector IncE
Source: Signal Transduct Target Ther. 2017 Jun 30;2:17030–. doi: 10.1038/sigtrans.2017.30 (PMC5661634; doi:10.1038/sigtrans.2017.30)
Supplement: Supplementary Figure S4 [file sigtrans201730-s5.pdf]

```
MOUSE_SNX5        SVSVDLNV-DPSLQIDIPDAL----SERDKVKFTVHTKTTLSTFQSPEFSVTRQHEDFVW   74
HUMAN_SNX5        SVSVDLNV-DPSLQIDIPDAL----SERDKVKFTVHTKTTLPTFQSPEFSVTRQHEDFVW
HUMAN_SNX6        AINVDLQS-DAALQVDISDAL----SERDKVKFTVHTKSSLPNFKQNEFSVVRQHEEFIW
HUMAN_SNX32       CASVDLQG-DSSLQVEISDAV----SERDKVKFTVQTKSCLPHFAQTEFSVVRQHEEFIW
ZEBRAFISH_SNX5    SVSVDLNN-DASLLIDIPDAL----CERDKVKFTVHTKTTLSPFQKPDFSVPRQHEDFIW
ZEBRAFISH_SNX6    AVNVDLQT-DATLQVDISDAL----SERDKVKFTVHTKSTLPNFKQNEFSVVRQHEEFIW
C.ELEGANS_SNX5    -----MNS-DEAICVDISDAL----SEREKVKYTVHTRTRLQEM-KPETAVVREHEEFLW
C.ELEGANS_SNX6    EINIDMNS-DEAICVDISDAL----SEREKVKYTVHTRTRLQEM-KPETAVVREHEEFLW
HUMAN_SNX1        ---EEEQEDQFDLTVGITDPEKIGDGMNAYVAYKVTTQTSLPLFRSKQFAVKRRFSDFLG
HUMAN_SNX2        ---EEANGDIFDIEIGVSDPEKVGDGMNAYMAYRVTTKTSLSMFSKSEFSVKRRFSDFLG
HUMAN_SNX3        NLNDAYGPPSNFLEIDVSNPQTVGVGRGRFTTYEIRVKTNLPIFKLKESTVRRRYSDFEW
HUMAN_SNX27       QSFYDYTE-KQAVPISVPRYKHVEQNGEKFVVYNVY--------MAGRQLCSKRYREFAI
                              : : :               : :                 :.. :*

MOUSE_SNX5        LHDTLTETTDYAGLIIPPAPTKPDFDGPREKMQKLGEGEGSMTKEEFAKMKQELEAEYLA   134
HUMAN_SNX5        LHDTLIETTDYAGLIIPPAPTKPDFDGPREKMQKLGEGEGSMTKEEFAKMKQELEAEYLA
HUMAN_SNX6        LHDSFVENEDYAGYIIPPAPPRPDFDASREKLQKLGEGEGSMTKEEFTKMKQELEAEYLA
HUMAN_SNX32       LHDAYVENEEYAGLIIPPAPPRPDFEASREKLQKLGEGDSSVTREEFAKMKQELEAEYLA
ZEBRAFISH_SNX5    LHDAIVETEEYAGLIIPPAPPKPDFEGPREKMHKLGEGESSMTKEEYAKMKQELEAEYLA
ZEBRAFISH_SNX6    LHDSFVENEEYAGYIIPPAPPRPDFDASREKLQKLGEGEGSMTKEEFTKMKQELEAEYLA
C.ELEGANS_SNX5    LHGTLEDNENYAGFIIPPAPPKPNFDSSREKLQKLGEGEATMTKEEFLKMKHDLEQDYLA
C.ELEGANS_SNX6    LHGTLEDNENYAGFIIPPAPPKPNFDSSREKLQKLGEGEATMTKEEFLKMKHDLEQDYLA
HUMAN_SNX1        LYEKLSEKHSQNGFIVPPPPEKSLIGMT-----KVKVGKEDSSSAEFL------------
HUMAN_SNX2        LHSKLASKYLHVGYIVPPAPEKSIVGMT-----KVKVGKEDSSSTEFV------------
HUMAN_SNX3        LRSELERE---SKVVVPPLPGKAFLRQL-----PF-RGDDGIFDDNFI------------
HUMAN_SNX27       LHQNLKR--EFANFTFPRLPGKWPFSLSEQQLDA--------------------------
                  *              .*  * :  .

MOUSE_SNX5        VFKKTVSTHEVFLQRLSSHPVLSKDRNFHVFLEYDQDLSVRRKNTK---              180
HUMAN_SNX5        VFKKTVSSHEVFLQRLSSHPVLSKDRNFHVFLEYDQDLSVRRKNTK---
HUMAN_SNX6        IFKKTVAMHEVFLCRVAAHPILRRDLNFHVFLEYNQDLSVRGKNKK---
HUMAN_SNX32       IFKKTVAMHEVFLQRLAAHPTLRRDHNFFVFLEYGQDLSVRGKNRK---
ZEBRAFISH_SNX5    VFKKTVQVHEVFLQRLSSHPSFSKDRNFHIFLEYDQDLSVRRKNAK---
ZEBRAFISH_SNX6    IFKKTVAMHEVFLCRVAAHPVLRKDLNFHVFLEYNQDLSVRGKNKK---
C.ELEGANS_SNX5    QFKKTVAMHEVFLQRIAAHPVFKNDQNFRIFLQYENELSVRGKNKK---
C.ELEGANS_SNX6    QFKKTVAMHEVFLQRIAAHPVFKNDQNFRIFLQYENELSVRGKNKK---
HUMAN_SNX1        --EKRRAALERYLQRIVNHPTMLQDPDVREFLEKEELPRAVGTQ-----
HUMAN_SNX2        --EKRRAALERYLQRTVKHPTLLQDPDLRQFLESSELPRAVNTQ-----
HUMAN_SNX3        --EERKQGLEQFINKVAGHPLAQNERCLHMFLQDEIIDKSYTPSKIRHA
HUMAN_SNX27       ----RRRGLEEYLEKVCSIRVIGESDIMQEFLSESDENY-NGVSDV---
                           * :: :        ..  .  **.          .
```

Figure S4
